# Polytrauma Defined by the New Berlin Definition: A Validation Test Based on Propensity-Score Matching Approach

**DOI:** 10.3390/ijerph14091045

**Published:** 2017-09-11

**Authors:** Cheng-Shyuan Rau, Shao-Chun Wu, Pao-Jen Kuo, Yi-Chun Chen, Peng-Chen Chien, Hsiao-Yun Hsieh, Ching-Hua Hsieh

**Affiliations:** 1 Department of Neurosurgery, Kaohsiung Chang Gung Memorial Hospital and Chang Gung University College of Medicine, No.123, Ta-Pei Road, Niao-Song District, Kaohsiung City 833, Taiwan; ersh2127@cloud.cgmh.org.tw; 2 Department of Anesthesiology, Kaohsiung Chang Gung Memorial Hospital and Chang Gung University College of Medicine, No.123, Ta-Pei Road, Niao-Song District, Kaohsiung City 833, Taiwan; shaochunwu@gmail.com; 3 Department of Plastic Surgery, Kaohsiung Chang Gung Memorial Hospital and Chang Gung University College of Medicine, No.123, Ta-Pei Road, Niao-Song District, Kaohsiung City 833, Taiwan; bow110470@gmail.com (P.-J.K.); libe320@yahoo.com.tw (Y.-C.C.); VENU_CHIEN@hotmail.com (P.-C.C.); sylvia19870714@hotmail.com (H.-Y.H.)

**Keywords:** polytrauma, new Berlin definition, Abbreviated Injury Scale (AIS), Glasgow Coma Scale (GCS), Injury Severity Score (ISS)

## Abstract

**Background:** Polytrauma patients are expected to have a higher risk of mortality than that obtained by the summation of expected mortality owing to their individual injuries. This study was designed to investigate the outcome of patients with polytrauma, which was defined using the new Berlin definition, as cases with an Abbreviated Injury Scale (AIS) ≥ 3 for two or more different body regions and one or more additional variables from five physiologic parameters (hypotension [systolic blood pressure ≤ 90 mmHg], unconsciousness [Glasgow Coma Scale score ≤ 8], acidosis [base excess ≤ −6.0], coagulopathy [partial thromboplastin time ≥ 40 s or international normalized ratio ≥ 1.4], and age [≥70 years]). **Methods:** We retrieved detailed data on 369 polytrauma patients and 1260 non-polytrauma patients with an overall Injury Severity Score (ISS) ≥ 18 who were hospitalized between 1 January 2009 and 31 December 2015 for the treatment of all traumatic injuries, from the Trauma Registry System at a level I trauma center. Patients with burn injury or incomplete registered data were excluded. Categorical data were compared with two-sided Fisher exact or Pearson chi-square tests. The unpaired Student *t*-test and the Mann–Whitney *U*-test was used to analyze normally distributed continuous data and non-normally distributed data, respectively. Propensity-score matched cohort in a 1:1 ratio was allocated using the NCSS software with logistic regression to evaluate the effect of polytrauma on patient outcomes. **Results:** The polytrauma patients had a significantly higher ISS than non-polytrauma patients (median (interquartile range Q1–Q3), 29 (22–36) vs. 24 (20–25), respectively; *p* < 0.001). Polytrauma patients had a 1.9-fold higher odds of mortality than non-polytrauma patients (95% CI 1.38–2.49; *p* < 0.001). Compared to non-polytrauma patients, polytrauma patients had a substantially longer hospital length of stay (LOS). In addition, a higher proportion of polytrauma patients were admitted to the intensive care unit (ICU), spent longer LOS in the ICU, and had significantly higher total medical expenses. Among 201 selected propensity score-matched pairs of polytrauma and non-polytrauma patients who showed no significant difference in sex, age, co-morbidity, AIS ≥ 3, and Injury Severity Score (ISS), the polytrauma patients had a significantly higher mortality rate (OR 17.5, 95% CI 4.21–72.76; *p* < 0.001), and a higher proportion of patients admitted to the ICU (84.1% vs. 74.1%, respectively; *p* = 0.013) with longer stays in the ICU (10.3 days vs. 7.5 days, respectively; *p* = 0.003). The total medical expenses for polytrauma patients were 35.1% higher than those of non-polytrauma patients. However, there was no significant difference in the LOS between polytrauma and non-polytrauma patients (21.1 days vs. 19.8 days, respectively; *p* = 0.399). **Conclusions:** The findings of this propensity-score matching study suggest that the new Berlin definition of polytrauma is feasible and applicable for trauma patients.

## 1. Background

The term “polytrauma” has been frequently defined in terms of a high Injury Severity Score (ISS) and has been generally used interchangeably with terms such as “severely injured” or “multiple trauma” [1]. The internationally accepted threshold of an ISS ≥ 16 is based on the description as being predictive of a mortality risk above 10% [2]. However, a number of definitions of polytrauma with various ISS values (ISS > 15 [3], ISS > 16 [4], ISS > 18 [5], ISS ≥ 18 [6], or ISS > 25 [7]) have been reported in the literature. In addition, a high ISS may be attributed to a severe single-system injury (monotrauma) rather than “polytrauma,” which refers to trauma patients whose injuries involve multiple body regions and in whom the combination of injuries would cause a life-threatening condition [8,9]. If this concept is considered, polytrauma patients are expected to have a higher mortality rate than that obtained by the summation of expected mortality owing to their individual injuries [10], and to have more expensive therapeutic requirements [11], to require intensive resources for resuscitation, and to stay longer in the intensive care unit (ICU) [10].

Without a clear definition of polytrauma, any attempt to compare the loads, interventions, and outcomes of polytrauma patients among various trauma centers is challenging [10]. Some authors have suggested that at least two anatomical regions have to be injured for a patient to be identified as having polytrauma [9,12,13,14]. The ‘polytrauma’ definition of Butcher and colleagues using the Abbreviated Injury Scale (AIS) ≥ 3 for at least two different body regions seemed more reasonable and feasible for identifying polytrauma patients [1,10,14]. With a higher mortality, more frequent ICU admissions, and longer hospital and ICU stays, this definition acts as a better predictor of morbidity and mortality than the definition using an ISS > 15 or ISS > 17 [1,10,14].

However, the definition of polytrauma based on the number of injured body regions does not reflect the physiological course after injury, which can be very dynamic in nature and may profoundly influence outcomes. Paffrath et al. even had reported that the mortality rate of polytrauma patients with an AIS ≥ 3 for at least two different body regions was even lower (18.7%) than that of patients with an ISS ≥ 16 (20.4%) [11]. Our unpublished study also revealed that under the definition of polytrauma by AIS ≥ 3 for at least two body regions, there was no significant difference in short-term mortality between polytrauma and non-polytrauma patients—i.e., polytrauma, as defined by AIS ≥ 3 for at least two body regions, was not a distinguishing factor for recognizing a significant difference in short-term mortality among trauma patients. To improve the specificity of the polytrauma definition, some additional qualifying criteria have been proposed, such as the requirement of laparotomy [15], existence of severe shock [16], involvement of at least one vital organ necessitating admission into the ICU [17], and systemic inflammatory response syndrome on at least one day during the first 72 h [10]. However, these additional criteria seemed to be limited and unverified. Moreover, the levels of variation and indication may differ among trauma centers.

The addition of a relevant physiologic condition or pathophysiologic change in combination of AIS/ISS is reasonable to increase its predictive power for mortality. Age, systolic blood pressure (SBP) and Glasgow Coma Scale (GCS) have been reported to have good predictive power for mortality [18]. An international consensus meeting in 2012 first tried to define polytrauma by combining the concept of injuries in different body regions and parameters of physiological response [19,20]. With the addition of at least one of five standardized physiological responses (hypotension [SBP ≤ 90 mmHg], unconsciousness [GCS score ≤ 8], acidosis [base excess ≤ −6.0], coagulopathy [partial thromboplastin time ≥ 40 s or international normalized ratio ≥ 1.4], and age [≥70 years]) in this new “Berlin definition” to the definition of ISS ≥ 16 and AIS ≥ 3 for at least two body regions, an improved definition of polytrauma was determined [13]. Notably, in the study that defines polytrauma as AIS ≥ 3 points for two or more different body regions, mortality was 11.4% and 11.0% in polytrauma and non-polytrauma patients, respectively. A mortality rate of 18.7% was found when polytrauma was defined using ISS ≥ 16 [13] and the mortality rates were increased to as high as 35–38% as soon as one other physiologic parameter was added [13].

Before applying the definition of the polytrauma in the clinical setting, we designed this study to investigate the outcome of polytrauma patients, with polytrauma being defined by the new Berlin definition, who admitted and treated for all trauma injuries at a level I trauma center. The primary hypothesis of this study was that polytrauma patients have a worse outcome than patients with similar injury severity but without polytrauma. In this study—for the assessment of the effect of polytrauma on the outcomes—we compared the selected propensity score-matched groups of patients to minimize confounding effects due to non-randomized assignment of patients into the polytrauma or non-polytrauma groups.

## 2. Methods

### Study Design

This study was approved by the Institutional Review Board (IRB) of Kaohsiung Chang Gung Memorial Hospital (reference number 201600544B0), a level I regional trauma center providing care to trauma patients primarily from southern Taiwan [21,22]. Informed consent was waived according to IRB regulations. This retrospective study reviewed data of all hospitalized patients registered in the Trauma Registry System from 1 January 2009 to 31 December 2015. All patients with an overall ISS ≥ 18 who were admitted for treatment of traumatic injuries were included and allocated into a polytrauma group or non-polytrauma group. The choice of 18 as a threshold of ISS for non-polytrauma patients depends on the consideration that the polytrauma patients at least would have an ISS of 18 (3^2^ + 3^2^ = 18). The new Berlin definition of polytrauma was used and defined as follows [13]: a patient with AIS ≥ 3 for two or more different body regions with additional one or more variables from the five physiologic parameters, including SBP ≤ 90 mm Hg, GCS score ≤ 8, base excess ≤ 6.0, international normalized ratio ≥ 1.4 or partial thromboplastin time ≥ 40 s, and age ≥ 70 years. 

The patients who had an overall ISS ≥ 18 but did not fit into the above criteria of polytrauma were defined as non-polytrauma patients. Patients with burn injury or incomplete registered data were excluded. Detailed patient information retrieved from the Trauma Registry System included the following: age; gender; trauma mechanism; initial GCS score in the emergency department (ED); vital signs assessed by the physician upon arrival at the ED and procedures performed by the physician at the ED (cardiopulmonary resuscitation, intubation, insertion of chest tube, and blood transfusion); co-morbidities, such as diabetes mellitus (DM), hypertension (HTN), coronary artery disease (CAD), congestive heart failure (CHF), cerebral vascular accident (CVA), and end-stage renal disease (ESRD); AIS severity score for each body region; ISS; rates of associated injuries; hospital length of stay (LOS); the rates of admission into the ICU as well as the LOS in ICU; in-hospital mortality; and total medical expenses, which included the cost of examination (physical examination, radiography examination, hematology testing, pathological examination, electrocardiography examination, endoscopy, echocardiogram, electromyography, cardiac catheterization, and electroencephalography monitoring), cost of operation (operation fee and operation supply fee), cost of pharmaceuticals (medical service, narcotics, and medicine), and other costs (fees for administrative tasks, registration, wards, nursing, blood/plasma tests, anesthesia, hemodialysis, rehabilitation, special material costs, and personal expenses), expressed as cost per victim in US dollars. The ISS is expressed as the median and interquartile range (IQR). Odds ratios (ORs) of the associated conditions and injuries of the patients were presented with 95% confidence intervals (CIs). The primary outcome of the study was in-hospital mortality, and the secondary outcomes were hospital LOS, ICU admission rate, ICU LOS, and the total medical expenses. The analysis was performed using IBM SPSS Statistics for Windows, version 20.0 (IBM Corp., Armonk, NY, USA). Two-sided Fisher exact or Pearson chi-square tests were used to compare categorical data. The unpaired Student *t*-test and Mann–Whitney *U*-test were used to analyze normally distributed continuous and non-normally distributed data, respectively, which was reported as mean ± standard deviation. To minimize confounding effects due to non-randomized assignment of patients into the polytrauma or non-polytrauma group, propensity score-matched groups of patients were selected for the assessment of the effect of polytrauma on the outcomes. A logistic regression model was used to calculate the propensity scores with the following covariates: gender; age, comorbidities, injury regions with AIS ≥ 3, and ISS. A 1:1 matched study group was created by the Greedy method with a 0.2 caliper width using NCSS 10 software (NCSS Statistical software, Kaysville, UT, USA). After adjustment of these confounding factors, binary logistic regression was used for evaluating the effect of polytrauma on the primary and secondary outcomes. *p*-values < 0.05 were considered statistically significant.

## 3. Results

### 3.1. Injury Characteristics and Severity of Polytrauma Patients

**Figure 1:**
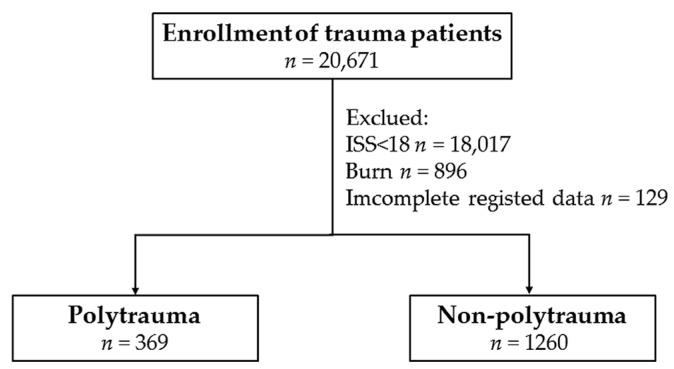
Flow chart of the studied polytrauma and non-polytrauma population.

After the exclusion of patients with an ISS less than 18 (*n* = 18,017), with a burn injury (*n* = 896), or incomplete registered data (*n* = 129) from 20,106 hospitalized patients, there were 369 and 1260 patients in the polytrauma and non-polytrauma group, respectively (Figure 1). No significant differences in sex, age, pre-existing commodities, and injury mechanism were found between the polytrauma and non-polytrauma patients (Table 1). GCS scores were significantly lower for polytrauma patients than for non-polytrauma patients (9.2 ± 4.6 vs. 11.8 ± 4.2, respectively; *p* < 0.001). Significantly more polytrauma patients had a GCS ≤ 8 than non-polytrauma patients. Analysis of injured body regions under the criteria of AIS ≥ 3, revealed that polytrauma patients had sustained significantly higher rates of face, thoracic, abdominal, and extremity injuries than non-polytrauma patients, while no difference in head and neck injury was found between polytrauma and non-polytrauma patients. The polytrauma patients had a significantly higher ISS than non-polytrauma patients (median (IQR: Q1–Q3), 29 (22–36) vs. 24 (20–25), respectively; *p* < 0.001). In addition, more polytrauma patients had an ISS ≥ 25 and fewer patients had an ISS of 18–24 as compared to non-polytrauma patients.

### 3.2. Outcomes of Polytrauma Patients

Polytrauma patients had 1.9-fold higher odds of mortality than non-polytrauma patients (95% CI 1.38–2.49; *p* < 0.001). Compared with non-polytrauma patients, polytrauma patients had significantly longer hospital LOS (20.0 days vs. 16.8 days, respectively; *p* < 0.001) and longer LOS in the ICU (10.1 days vs. 8.3 days, respectively; *p* = 0.005). Moreover, compared to non-polytrauma patents, a higher proportion of polytrauma patients were admitted to the ICU (80.8% vs. 75.5%, respectively; *p* = 0.035). In addition, the polytrauma patients spent a significantly higher amount on medical expenses (41.8% higher), examinations (43.8% higher), operations (35.3% higher), and pharmaceuticals (58.2% higher) than non-polytrauma patients.

### 3.3. Associated Management and Injuries of Polytrauma Patients

Polytrauma patients had significantly higher odds for worse hemodynamic measures and the requirement of procedures at the ED than non-polytrauma patients (Table 2). These measures included an SBP of <90 mmHg, heart rate of >100 beats/min, and respiratory rate of <10 or >29 times/min. The required procedures included cardiopulmonary resuscitation, intubation, insertion of chest tube, and blood transfusion. Polytrauma patients had significantly higher ORs for sustaining subarachnoid hemorrhage (OR 1.4, 95% CI 1.08–1.73; *p* = 0.010) but lower ORs for subdural hematomas (OR 0.7, 95% CI 0.57–0.91; *p* = 0.006) than non-polytrauma patients (Table 3). In addition, polytrauma patients had significantly higher ORs for sustaining trauma in the thoracic, abdominal, and extremity regions than non-polytrauma patients.

### 3.4. Adjusted Outcomes of Polytrauma Patients in Propensity Score–Matched Patient Population

A propensity score–matched patient population was selected to reduce the impact of demographic differences, pre-existing co-morbidities, and injury severity of the patient population on the outcome assessment between polytrauma and non-polytrauma patients (Table 4). In these 201 selected, well-balanced pairs of patients, there were no significant differences in sex, age, co-morbidity, number of patients with AIS ≥ 3, and ISS. The logistic regression analysis of these pairs of patients showed that polytrauma patients had significantly higher mortality (OR 17.5, 95% CI 4.21–72.76; *p* < 0.001) than non-polytrauma patients. Compared with non-polytrauma patients, the polytrauma patients had a higher proportion of patients admitted to the ICU (84.1% vs. 74.1%, respectively; *p* = 0.013) and a longer stay in ICU (10.3 days vs. 7.5 days, respectively; *p* = 0.003), but there was no significant difference in the hospital LOS (21.1 days vs. 19.8 days, respectively; *p* = 0.399). In addition, polytrauma patients still had a significantly higher total medical expense (35.1% higher), cost of examination (33.1% higher), cost of operation (40.6% higher), and cost of pharmaceuticals (53.9% higher) than the non-polytrauma patients.

## 4. Discussion

This study compared clinical outcomes in a broad group of hospitalized polytrauma and non-polytrauma patients to investigate the feasibility and applicability of the new Berlin definition of polytrauma. We found that polytrauma patients presented with significantly higher morbidity and mortality than non-polytrauma patients. More importantly, even after consideration of the differences in demographic characteristics, co-morbidities, and injury severity of the trauma patient population, the selected propensity score–matched polytrauma patients still had a significantly higher proportion of patients admitted to the ICU with a longer stay, a higher total medical cost, and higher mortality than non-polytrauma patients.

Polytrauma is generally used to describe trauma patients whose injuries involve multiple body regions, compromise the patient’s physiology and potentially cause dysfunction of uninjured organs [13]. The expected higher risk of mortality of polytrauma patients is based on the assumption that the underlying pathophysiological response of the injured person would aggravate the clinical outcome. The injured person’s pathophysiological response to the injury load, however, makes a differentiation between “polytrauma” and “multitrauma” [10]. For example, many reports had indicated that head and brain injuries and thoracic traumata are major risk factors in trauma patients and that co-occurrence of these factors leads to an exponential increase in mortality [23,24,25]. The reduced pulmonary reserves in lung contusion may rapidly lead to hypoventilation and hypoxia that cause secondary injury to the brain or pose more burden to other organ systems. Early intubation was also a significant risk factor for cerebral impairment in patients with multiple trauma [26]. The injury to the gastrointestinal system may interfere with the nutrition balance and increase bacterial translocation [27,28]—moreover, increasing levels of endotoxemia following polytrauma have been reported [29]. Likewise, hypovolemic shock and massive blood transfusion are often associated with coagulopathy and imbalance in acid-base homeostasis [30]. Notably, in this study polytrauma patients had statistically significant higher ORs for sustaining thoracic trauma and abdominal trauma as well as for requiring procedures including cardiopulmonary resuscitation, intubation, chest tube insertion, and blood transfusion at the ED. These combined scenarios may explain partly, but not enough, our observation of a higher mortality in the selected propensity score–matched polytrauma patients than that in non-polytrauma patients.

A combination of injury severity, relevant pathophysiologic change, or physiologic changes seemed be useful for mortality prediction [13]. The dysregulation of the immune system after trauma presents one of the greatest threats to life [31,32]. However, a formally defined pathophysiological response to trauma remains a challenge. In addition, even in a prospective study, the practicability of including systemic inflammatory response syndrome into the definition of polytrauma as a surrogate for physiological derangement was questioned [33]. In contrast, the selected five physiological parameters (hypotension [SBP ≤ 90 mmHg], unconsciousness [GCS score ≤ 8], acidosis [base excess ≤ −6.0], coagulopathy [partial thromboplastin time ≥ 40 s or international normalized ratio ≥ 1.4], and age [≥70 years]) of the new “Berlin definition” for polytrauma are deemed feasible worldwide and easily approachable with less ambiguity [13]. This study based on a propensity-score matching approach may provide more evidence to support the use of such a definition of polytrauma in clinical settings to manage with polytrauma patients.

In this study, the non-polytrauma group is composed of two different types of patients: first, patients with only one body region affected (AIS ≥ 4), with or without physiological problems; and second, patients with two or more body regions affected, but no physiological problem. The matching used in this study is prone to select the latter group of patients for comparison, thus may have a selection bias. This study has some other limitations that should be acknowledged. First, the retrospective design of the study would carry an inherent selection bias. Second, the patients declared dead at the accident scene or on hospital arrival were not included in the Trauma Registry Database, which may have led to a selection bias. Third, as the registered data lacked uniform indication of hospitalization and admission into ICU, as well as the type of surgery performed on patients, we could only rely on the assumption that management of patients with or without polytrauma was uniform. Further, long-term mortality was not evaluated in this study. Finally, the physiological problems also work in isolated trauma [11]. With an increasing number of physiological factors there was an almost linear increase in mortality up to an 86% rate in patients with all five physiological factors present [11]. In this study, the addition of physiological factor(s) to the polytrauma but not to non-polytrauma patients, making the present non-polytrauma group is a mixture of two groups with separate injury pattern, i.e. one with, and one without, a physiological problem. Therefore, a selection bias may be existed in the comparison.

## 5. Conclusions

As we noted a significantly higher incidence of morbidity and mortality in polytrauma patients than in non-polytrauma patients, our study findings based on propensity-score matching validate the new definition of polytrauma based on the new Berlin definition, as a case with AIS ≥ 3 for two or more different body regions with additional one or more variables from the five physiologic parameters.

## Figures and Tables

**Table 1:** Demographics and injury characteristics of polytrauma and non-polytrauma patients.

Variables	Polytrauma *n* = 369	Non-Polytrauma *n* = 1260	*Odds Ratio (95% CI)*	*p*
Sex				0.610
Male	255 (69.1)	853 (67.7)	1.1 (0.83–1.37)	
Female	114 (30.9)	407 (32.3)	0.9 (0.73–1.20)	
Age	49.9 ± 22.9	47.3 ± 19.9	—	0.052
Comorbidity				
DM	37 (10.0)	169 (13.4)	0.7 (0.49–1.05)	0.085
HTN	79 (21.4)	275 (21.8)	1.0 (0.74–1.29)	0.865
CAD	15 (4.1)	36 (2.9)	1.4 (0.78–2.66)	0.241
CHF	1 (0.3)	3 (0.2)	1.1 (0.12–10.98)	1.000
CVA	5 (1.4)	30 (2.4)	0.6 (0.22–1.46)	0.232
ESRD	0 (0.0)	1 (0.1)	—	1.000
Mechanism, n (%)				
MV Driver	19 (5.1)	39 (3.1)	1.7 (0.97–2.98)	0.061
MV Passenger	11 (3.0)	19 (1.5)	2.0 (0.95–4.26)	0.064
Motorcycle Driver	222 (60.2)	792 (62.9)	0.9 (0.70–1.13)	0.348
Motorcycle Pillion	18 (4.9)	38 (3.0)	1.6 (0.93–2.93)	0.084
Bicycle	10 (2.7)	53 (4.2)	0.6 (0.32–1.26)	0.190
Pedestrian	20 (5.4)	48 (3.8)	1.4 (0.85–2.47)	0.174
Fall	59 (16.0)	230 (18.3)	0.9 (0.62–1.17)	0.317
Penetrating injury	1 (0.3)	2 (0.2)	1.7 (0.16–18.90)	0.537
Struck by/against	9 (2.4)	39 (3.1)	0.8 (0.38–1.63)	0.512
GCS	9.2 ± 4.6	11.8 ± 4.2	—	<0.001
≤8	200 (54.2)	301 (23.9)	3.8 (2.96–4.80)	<0.001
9-12	32 (8.7)	181 (14.4)	0.6 (0.38–0.84)	0.004
≥13	137 (37.1)	778 (61.7)	0.4 (0.29–0.47)	<0.001
AIS ≥ 3, n (%)				
Head/Neck	281 (76.2)	940 (74.6)	1.1 (0.83–1.43)	0.546
Face	19 (5.1)	12 (1.0)	5.6 (2.71–11.74)	<0.001
Thorax	237 (64.2)	378 (30.0)	4.2 (3.28–5.35)	<0.001
Abdomen	82 (22.2)	136 (10.8)	2.4 (1.74–3.20)	<0.001
Extremity	183 (49.6)	191 (15.2)	5.5 (4.26–7.11)	<0.001
ISS, median (IQR)	29 (22–36)	24 (20–25)	—	<0.001
18–24	100 (27.1)	727 (57.7)	0.3 (0.21–0.35)	<0.001
≥25	269 (72.9)	533 (42.3)	3.7 (2.84–4.74)	<0.001
Mortality, n (%)	80 (21.7)	164 (13.0)	1.9 (1.38–2.49)	<0.001
Hospital LOS (days)	20.0 ± 16.8	16.8 ± 15.6	—	0.001
ICU				
Patients, n (%)	298 (80.8)	951 (75.5)	1.4 (1.02–1.82)	0.035
18–24	66 (22.1)	479 (50.4)	0.3 (0.21–0.38)	<0.001
≥25	232 (77.9)	472 (49.6)	3.6 (2.64–4.82)	<0.001
LOS in ICU (days)	10.1 ± 9.5	8.3 ± 9.5	—	0.005
Medical expenses	8888 ± 8141	6270 ± 6915	—	<0.001
Cost of examination	591 ± 536	411 ± 493	—	<0.001
Cost of operation	1103 ± 1198	815 ± 1084	—	<0.001
Cost of pharmaceuticals	791 ± 1291	500 ± 1028	—	<0.001

AIS = abbreviated injury scale; CAD = coronary artery disease; CHF = Congestive Heart Failure; CI = confidence interval; CVA = cerebral vascular accident; DM = diabetes mellitus; ESRD = end-stage renal disease; GCS = Glasgow Coma Scale; HTN = hypertension; ICU = intensive care unit; IQR = interquartile range; ISS = injury severity score; LOS = length of stay; MV = motor vehicle.

**Table 2:** Physiological response and procedures performed upon arrival at the emergency department for polytrauma and non-polytrauma patients.

Variables	Polytrauma *n* = 369	Non-Polytrauma *n* = 1260	*Odds Ratio (95% CI)*	*p*
Physiology at ED, n (%)				
SBP < 90 mmHg	80 (21.7)	50 (4.0)	6.7 (4.60–9.76)	<0.001
HR > 100 beats/min	154 (41.7)	358 (28.4)	1.8 (1.42–2.30)	<0.001
RR < 10 or > 29 times/min	36 (9.8)	29 (2.3)	4.6 (2.77–7.60)	<0.001
Procedures at ED, n (%)				
Cardiopulmonary resuscitation	15 (4.1)	11 (0.9)	4.8 (2.19–10.57)	<0.001
Intubation	90 (24.4)	187 (14.8)	1.9 (1.39–2.46)	<0.001
Chest tube insertion	59 (16.0)	84 (6.7)	2.7 (1.87–3.80)	<0.001
Blood transfusion	145 (39.3)	132 (10.5)	5.5 (4.20–7.29)	<0.001

ED = emergency department; HR = heart rate; RR = respiratory rate; SBP = systolic blood pressure.

**Table 3:** Significant associated injuries in polytrauma and non-polytrauma patients.

Variables	Polytrauma *n* = 369	Non-Polytrauma *n* = 1260	*Odds Ratio (95% CI)*	*p*
Head trauma, n (%)				
Neurologic deficit	14 (3.8)	33 (2.6)	1.5 (0.78–2.77)	0.236
Cranial fracture	100 (27.1)	349 (27.7)	1.0 (0.75–1.26)	0.821
Epidural hematoma (EDH)	74 (20.1)	310 (24.6)	0.8 (0.58–1.02)	0.070
Subdural hematoma (SDH)	155 (42.0)	631 (50.1)	0.7 (0.57–0.91)	0.006
Subarachnoid hemorrhage (SAH)	157 (42.5)	443 (35.2)	1.4 (1.08–1.73)	0.010
Intracerebral hematoma (ICH)	46 (12.5)	129 (10.2)	1.2 (0.87–1.79)	0.224
Cerebral contusion	71 (19.2)	261 (20.7)	0.9 (0.68–1.22)	0.537
Maxillofacial trauma, n (%)				
Orbital fracture	10 (2.7)	55 (4.4)	0.6 (0.31–1.21)	0.153
Maxillary fracture	53 (14.4)	195 (15.5)	0.9 (0.66–1.27)	0.601
Mandibular fracture	15 (4.1)	48 (3.8)	1.1 (0.59–1.93)	0.823
Thoracic trauma, n (%)				
Rib fracture	141 (38.2)	337 (26.7)	1.7 (1.33–2.16)	<0.001
Hemothorax	59 (16.0)	89 (7.1)	2.5 (1.76–3.56)	<0.001
Pneumothorax	50 (13.6)	107 (8.5)	1.7 (1.18–2.42)	0.004
Hemopneumothorax	53 (14.4)	92 (7.3)	2.1 (1.49–3.05)	<0.001
Lung contusion	48 (13.0)	72 (5.7)	2.5 (1.68–3.63)	<0.001
Abdominal trauma, n (%)				
Hepatic injury	57 (15.4)	103 (8.2)	2.1 (1.45–2.90)	<0.001
Splenic injury	46 (12.5)	47 (3.7)	3.7 (2.40–5.62)	<0.001
Retroperitoneal injury	9 (2.4)	8 (0.6)	3.9 (1.50–10.21)	0.006
Renal injury	13 (3.5)	25 (2.0)	1.8 (0.91–3.56)	0.085
Extremity trauma, n (%)				
Clavicle fracture	54 (14.6)	224 (17.8)	0.8 (0.57–1.10)	0.158
Humeral fracture	26 (7.0)	54 (4.3)	1.7 (1.04–2.74)	0.031
Radial fracture	38 (10.3)	78 (6.2)	1.7 (1.16–2.61)	0.007
Ulnar fracture	22 (6.0)	54 (4.3)	1.4 (0.85–2.36)	0.179
Pelvic fracture	43 (11.7)	82 (6.5)	1.9 (1.28–2.80)	0.001
Femoral fracture	99 (26.8)	86 (6.8)	5.0 (3.64–6.88)	<0.001
Patella fracture	15 (4.1)	18 (1.4)	2.9 (1.46–5.86)	0.002
Tibia fracture	58 (15.7)	59 (4.7)	3.8 (2.59–5.57)	<0.001
Fibular fracture	43 (11.7)	44 (3.5)	3.6 (2.35–5.65)	<0.001

**Table 4:** Covariates and the assessment of outcomes in polytrauma and non-polytrauma patients adjusted in 1:1 Greedy propensity-score matching.

Variables	Polytrauma *n* = 201	Non-Polytrauma *n* = 201	*Odds Ratio (95%)*	*p*
Sex				1.000
Male	149 (74.1)	149 (74.1)	1.0 (0.64–1.56)	
Female	52 (25.9)	52 (25.9)	1.0 (0.64–1.56)	
Age	43.2 ± 20.0	43.4 ± 16.4	—	0.919
Comorbidity				
DM	12 (6.0)	12 (6.0)	1.0 (0.44–2.28)	1.000
HTN	27 (13.4)	27 (13.4)	1.0 (0.56–1.77)	1.000
CAD	0 (0.0)	0 (0.0)	—	—
CHF	0 (0.0)	0 (0.0)	—	—
CVA	0 (0.0)	0 (0.0)	—	—
ESRD	0 (0.0)	0 (0.0)	—	—
AIS ≥ 3, *n* (%)				
Head/Neck	144 (71.6)	144 (71.6)	1.0 (0.65–1.54)	1.000
Face	5 (2.5)	5 (2.5)	1.0 (0.29–3.51)	1.000
Thorax	133 (66.2)	133 (66.2)	1.0 (0.66–1.51)	1.000
Abdomen	45 (22.4)	45 (22.4)	1.0 (0.63–1.60)	1.000
Extremity	99 (49.3)	99 (49.3)	1.0 (0.68–1.48)	1.000
ISS, median (IQR)	27 (22–34)	26 (22–29)	—	0.271
Mortality, n (%)	35 (17.4)	2 (1.0)	17.5 (4.21–72.76)	<0.001
Hospital LOS (days)	21.1 ± 16.2	19.8 ± 14.4	—	0.399
ICU				
Patients, n (%)	169 (84.1)	149 (74.1)	2.0 (1.15–3.30)	0.013
LOS in ICU (days)	10.3 ± 9.3	7.5 ± 7.4	—	0.003
Medical expenses	9634 ± 8850	7129 ± 6800	—	0.002
Cost of examination	627 ± 584	471 ± 516	—	0.005
Cost of operation	1225 ± 1238	871 ± 1039	—	0.002
Cost of pharmaceutical	851 ± 1431	553 ± 1085	—	0.019

AIS = abbreviated injury scale; CAD = coronary artery disease; CHF = Congestive Heart Failure; CI = confidence interval; CVA = cerebral vascular accident; DM = diabetes mellitus; ESRD = end-stage renal disease; HTN = hypertension; ICU = intensive care unit; IQR = interquartile range; ISS = injury severity score; LOS = length of stay.
